# Post-Infectious Inflammatory Response Syndrome in an HIV-Negative Immunocompetent Elderly Patient With Cryptococcal Meningitis: A Case Report and Literature Review

**DOI:** 10.3389/fimmu.2022.823021

**Published:** 2022-02-23

**Authors:** Junyu Liu, Jia Liu, Bang-e Qin, Shiqi Yao, Anni Wang, Lu Yang, Zhihui Su, Xiaofeng Xu, Ying Jiang, Fuhua Peng

**Affiliations:** Department of Neurology, The Third Affiliated Hospital of Sun Yat-sen University, Guangzhou, China

**Keywords:** corticosteroids, cryptococcal meningitis (CM), elderly patients, post-infectious inflammatory response syndrome, cerebrospinal fluid, brain MRI

## Abstract

We report a previously healthy 82-year-old male with cryptococcal meningitis (CM) who represented neurological deterioration due to post-infectious inflammatory response syndrome (PIIRS) occurring in 4 months after initial antifungal therapy. He was treated with corticosteroids for 2 months and recovered clinically. However, the clinical manifestation, cerebrospinal fluid (CSF), and brain magnetic resonance imaging (MRI) results got worse again on the next day after corticosteroid withdrawal. The analysis of inflammatory cytokines and culture on CSF, as well as brain MRI, still suggested a diagnosis of PIIRS. Therefore, corticosteroid therapy was used again and he subsequently obtained a complete resolution of symptoms.

## Introduction

Cryptococcal meningitis (CM) is the most common adult fungal meningitis in large parts of the world with high morbidity and mortality (1–3). An exaggerated immunological response after starting antiretroviral therapy (ART) treatment was initially observed in HIV-infected patients with CM and termed as immune reconstitution inflammatory syndrome (IRIS) (4). In the HIV-negative CM patients, neurological deterioration is not due to antifungal treatment failure but due to the enhancement of immune response, which is called post-infectious inflammatory response syndrome (PIIRS) (5, 6). Here, we report an 82-year-old male patient with CM who developed PIIRS after initial antifungal therapy. To the best of our knowledge, CM-PIIRS had not been reported at such an old age.

### Case Presentation

**Figure 1:**
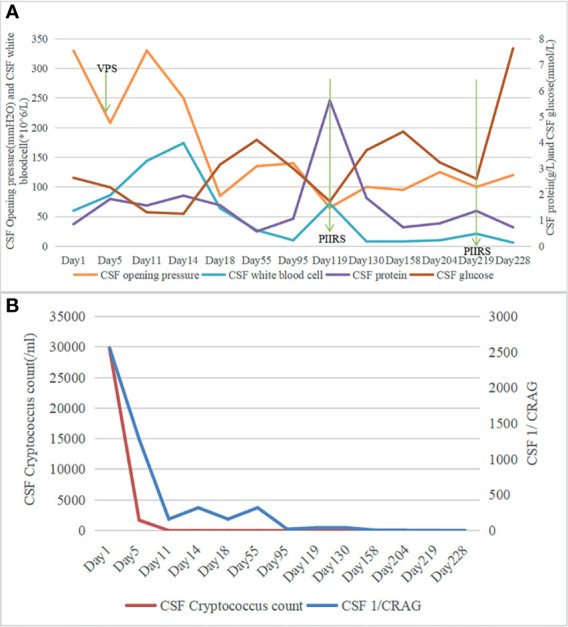
**(A)** Chronologic representation of serial cerebrospinal fluid (CSF) measurements obtained from lumbar puncture with CSF opening pressure and CSF white blood cell (WBC) count on the left axis and CSF protein, CSF glucose on the right axis. **(B)** Chronologic representation of serial CSF measurements obtained from lumbar puncture with CSF cryptococcus count on the left axis and CSF 1/CRAG on the right axis.

An 82-year-old man sought treatment for a 1-month history of headache and a 1-day history of altered mental status. He had a 10-year history of hypertension with irregular medication and no histories of head trauma, or surgery. Moreover, he had no histories of contacting with pigeons and other birds. On admission, his temperature was 36.5°C. Physical examination showed drowsiness, Glasgow score of 12, neck stiffness, and positive Kernig’s sign. Furthermore, he did not have gait abnormalities, cranial nerve deficits, or visual or hearing deficits. Serum HIV antibody was negative. Lumbar puncture (LP) (see **Figure 1**) showed a high opening pressure (OP) of 33 cm H_2_O, protein of 0.85 g/l, glucose of 2.64 mmol/l, and white blood cell (WBC) counts of 60 × 10^6^/l (with 30% neutrophils and 70% lymphocytes). Cerebrospinal fluid (CSF) India ink stain and culture were positive for *Cryptococcus neoformans*, and cryptococcal antigen titer (CRAG) in CSF was 1:2,560. Therefore, he was diagnosed with CM and treated immediately with amphotericin B deoxycholate plus 5-flucytosine. Due to the high intracranial pressure, a ventriculoperitoneal shunt (VPS) was placed on day 4 (note: day 1 represented admission day). After VPS, his CSF OP returned to 208 mmH_2_O and mental status improved (GCS score of 15). Three weeks after the initial antifungal therapy, his headache and neck stiffness disappeared, CSF culture was sterile, and CSF CRAG decreased to 1:160. Moreover, he was discharged with oral voriconazole and 5-flucytosine on day 26.

**Figure 2:**
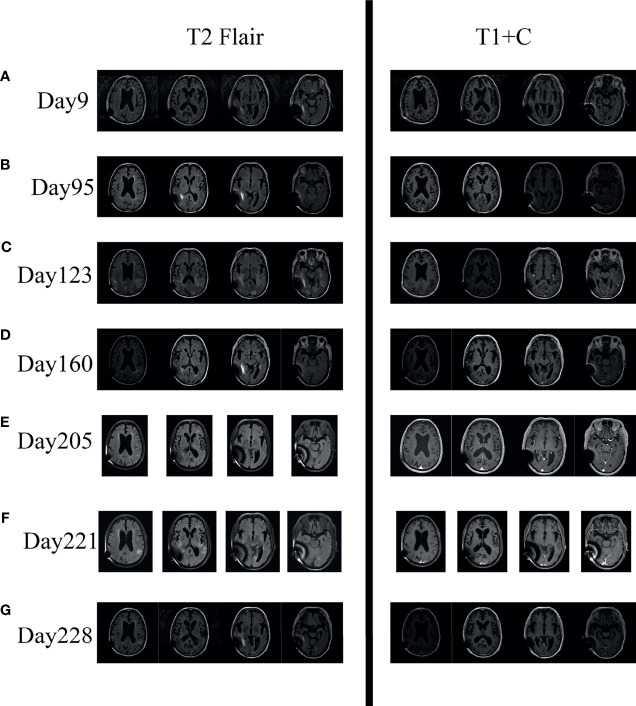
Serial axial brain MR imaging of the patient. **(A, B)** Imaging on Day 9 and Day 95 showed enlarged ventricles and widened brain fissure; T1+C showed no abnormal enhancement. **(C)** Imaging on Day 123 showed new lesions in bilateral occipital lobe, insular lobe, hippocampus, and paraventricular (T2 Flair); T1+C showed meningeal enhancement and ependymitis/choroiditis after effective antifungal therapy. **(D, E)** Imaging on Day 160 and Day 205 showed marked improvement of the lesions on day 123 after corticosteroid treatment. **(F)** Imaging on Day 221 showed that the lesions reappeared after discontinuing corticosteroid. **(G)** Imaging on Day 228 showed that the lesions decreased after reusing corticosteroid.

During the 3 months after discharge, he was asymptomatic. However, on day 118, he developed an unbearable headache. Physical examination revealed no neurological abnormalities. The laboratory results showed an elevated C-reactive protein of 14 mg/l (normal value <6 mg/l). LP (see **Figure 1**) showed OP of 65 mmH_2_0, WBC counts of 72 × 10^6^/l (67% lymphocytes), glucose of 1.70 mmol/l, protein of 5.62 g/l, and CRAG of 1:40. CSF India ink stain and culture were negative for *Cryptococcus neoformans*. Chest CT was unremarkable. Brain MRI on day 123 revealed meningeal enhancement, and new lesions in the bilateral occipital lobe, insular lobe, hippocampus, and paraventricular appeared (see **Figures 2A–C**). The PIIRS was considered, then he received intravenous dexamethasone (15 mg/day, 7 days) followed by oral methylprednisolone 28 mg daily (tapered by 4 mg every week). His headache disappeared on day 124, and repeated CSF examination on day 130 showed that the WBC count decreased to 8 × 10^6^/l, CSF India ink stain and cultures still were negative, and CSF CRAG was 1:40. Then, he was discharged with voriconazole and oral methylprednisolone.

Two months later (day 205), his methylprednisolone tablets reduced to 4 mg per day and he was asymptomatic, and the repeated brain MRI improved significantly (see **Figures 2C–E**). Therefore, corticosteroids were discontinued on day 206. However, only one day later, he developed headache, dizziness, and fever. Then, he was treated with antipyretic in the local hospital, but his symptoms did not improve.

Thirteen days later (day 219), he was readmitted to our hospital for intractable headache and fatigue. A repeated LP showed OP of 100 mmH_2_O, negative CSF India ink stain, culture, and CRAG. Moreover, the CSF tests for inflammatory cytokines demonstrated elevated levels of IL-6 (1,378.72 pg/ml, normal range: ≤18.6 pg/ml). Repeated brain MRI (day 221) showed the lesions that disappeared after corticosteroid treatment reappeared (see **Figure 2F**). PIIRS was still considered. To reduce robust inflammation, corticosteroid therapy was given again with 7 days of 20 mg intravenous dexamethasone which was slowly tapered. One week later (day 228), his clinical symptoms were relieved, CSF IL-6 level (8.12 pg/ml, normal range: ≤18.6 pg/ml) decreased, and brain MRI improved (see **Figure 2G**).

## Discussion

To the best of our knowledge, this is the oldest patient reported for CM-PIIRS. He developed PIIRS in 4 months after initial antifungal therapy. Then, he was treated with corticosteroids for 2 months and recovered clinically. However, the clinical manifestation, CSF, and brain MRI results got worse again on the next day after corticosteroid withdrawal. He received corticosteroids again and subsequently obtained a complete resolution of symptoms.

PIIRS is defined as a deterioration in neurological status in a previously healthy patient with CM after CSF fungal culture converting to negative following optimal therapy (7, 8). Unlike IRIS where immune reconstitution occurs after the initiation of ART, PIIRS may be a response to released fungal antigens during therapy and reductions in immunomodulatory components including capsular fragments (9). Although the exact reason for CM-PIIRS remains unclear, some studies have also been carried out. Our previous study indicated that baseline hearing impairment and high CSF pressure (>230 mmH2O) may be predictors of PIIRS in HIV-negative immunocompetent CM patients (10, 11), and a prospective study including 25 patients showed that 88% of non-HIV CM patients developed PIIRS after VPS (10, 11). As some literature reported (5, 6, 11–20), our case got initial neurological improvement after the use of corticosteroids. However, this case developed PIIRS again on the next day after 2 months of corticosteroid treatment withdrawal, and we have to think about the question: what dose of corticosteroids should we use and how long should we use corticosteroids in CM-PIIRS?

US guidelines suggested that 0.5–1.0 mg/kg per day of prednisone equivalent and 2–6 weeks of corticosteroid treatment were a reasonable method for patients with severe CM-IRIS (21), while in CM-PIIRS, there were no clinical guidelines to instruct clinicians how to use corticosteroids. As shown in **Table 1**, there are currently two common methods of using corticosteroids for treating CM-PIIRS: (1) pulse methylprednisolone (1 g IV daily for 5–7 days) taper therapy (6) and (2) a lower dose of corticosteroid (dexamethasone 10–20 mg/day IV or prednisolone 1 mg/kg/day PO) taper therapy. In a recent prospective study (6), CM-PIIRS patients received pulse methylprednisolone 1 g IV daily for 7 days followed by 1 mg/kg/day prednisone for 1 month and then tapered by 5 mg every month based on their clinical response and brain MRI findings. Romani et al. (13) described a CM-PIIRS patient with X-linked Hyper IgM syndrome who received 6 days of high-dose methylprednisolone (1 g) which was slowly tapered over 6 months. However, we treat CM-PIIRS patients with low-dose corticosteroid taper therapy in our department. As shown in our previous published study (14), CM-PIIRS patients were treated with intravenous dexamethasone (10–20 mg daily), then oral prednisone (30–40 mg/day), and tapered off by time. There are other reports on the use of low-dose corticosteroid taper therapy in CM-PIIRS (14–16, 18). For instance, Kulkarni et al. (18) reported a CM-PIIRS patient who received oral prednisolone (1 mg/kg/day) for 1 week and then tapered over 2 months, and this patient had significant clinical improvement and was discharged in a stable condition. The above two methods of using corticosteroids seem to be effective for CM-PIIRS, but further research is needed to evaluate which method is better.

**Table 1:** The course and dosage of corticosteroids for CM-PIIRS.

Reference	N	The course and dosage of corticosteroids	Outcome	Type of study
Romani et al. (13)	1	6 days of 1 g IV methylprednisolone, then corticosteroids (30 mg PO prednisone bid) were tapered over 6 months reducing 5 mg every week.	After 1 year of follow-up, no neurological sequelae were observed or reported.	Case report
Cheng et al. (15)	1	Dexamethasone was initiated at 0.3 mg/kg/day and tapered over a 2-week period with transition to oral steroids (totally 4 weeks) and restarted higher doses of steroids with a slower wean over the next 8 months (totally 8 months) and a short course of higher dose steroids followed by a prolonged taper (totally 9 months).	He was asymptomatic with normal MRI.	Case report
Yang et al. (11)	22	9 patients were injected with prednisone (0.75–1.5 mg/kg/day) or dexamethasone (10–20 mg/day) for 7–10 days and then gradually reduced oral low-dose prednisone for 7 days. 2 patients received prolonged prednisone treatment, which were 22 and 4 months, respectively.	7 patients improved only after receiving corticosteroids therapy.	Prospective and observational study
Anjum et al. (6)	15	Methylprednisolone 1 g IV daily for 7 days followed by 1 mg/kg/day prednisone for 1 month and were then tapered by 5 mg every month based on their clinical response and magnetic resonance imaging (MRI) brain findings.	All patients demonstrated significant improvements in MOCA and Karnofsky scores at 1 month, which was accompanied by improvements in CSF glucose, white blood cell count, protein, and cellular and soluble inflammatory markers 1 week after receiving corticosteroids.	Prospective, observational study
Liu et al. (10)	23	Intravenous dexamethasone (10–20 mg daily), then oral prednisone (30–40 mg/day) and then tapered off by time. Steroid therapy duration was 1–12 months (median 4 months).	Receipt of corticosteroid therapy was associated with lower rates of fever and better modified Rankin Score scores at 1 month after treatment.	Case–control study
Kathiresu et al. (17)	1	High-dose intravenous corticosteroids, followed by an oral course that was tapered over 6 months	The patient remarkably improved within the next week and returned to the ward for continuation of rehabilitation.	Case report
Tanu et al. (16)	1	Steroids (dexamethasone 12 mg/day) were initiated and tapered gradually for a total of 6 weeks. Prednisolone 20 mg/day) was given for another 2 weeks and slowly tapered over the next 3 months.	He got clinical improvement.	Case report
Kulkarni et al. (18)	1	Oral prednisolone (1 mg/kg/day) in tapering dosage continued for period of 2 months.	Defervescence of headache and significant neurological improvement was observed over a period of 7 days after commencing steroids.	Case report
Mehta et al. (19)	8	4 patients received IV methylprednisolone (1 g daily) or dexamethasone (12–15 mg daily), followed by maintenance therapy using oral prednisone (20 mg daily). In the 4 remaining patients, oral prednisone was used for both induction (60–90 mg daily) and maintenance therapy (20 mg daily). Corticosteroid therapy duration was 1–27 months (median 8 months).	At 1 month of corticosteroid therapy initiation, objective neurological improvements were noted in five patients (63%). No corticosteroid-related adverse events were noted.	Retrospective study

Our case showed the CM-PIIRS patient who got worse in clinical manifestation and brain imaging again just one day after corticosteroid withdrawal. The phenomenon seen in our case was also similar to previous studies (16, 20). One report (16) showed an immunocompetent 34-year-old CM patient who developed PIIRS. Dexamethasone 12 mg/day was initiated and tapered gradually and eventually stopped in 1 month. However, the patient experienced headache 4 days after corticosteroid withdrawal. Therefore, prednisolone (20 mg/day) was given for another 2 weeks and slowly tapered over the next 3 months with sustained clinical improvement. Moreover, another report (20) showed that CM-PIIRS occurred in a 46-year-old female when the steroids were discontinued; she received corticosteroids for more than 10 months in total. Therefore, how long should we use corticosteroids in CM-PIIRS? Our previous report (14) showed that the corticosteroid therapy median duration was 4 months and Mehta et al. (19) reported corticosteroid salvage therapy (CST), followed by maintenance therapy using oral prednisone (20 mg daily); the median duration was 8 months. In addition, Cheng et al. (15) reported a CM-PIIRS patient who received corticosteroids for more than 1 year and Kathiresu et al. (17) reported a CM-PIIRS patient who received corticosteroids for more than 6 months. Moreover, there were other reports that showed a more than 2-month (10, 6, 5 months, respectively) course of corticosteroids in CM-PIIRS patients (6, 13, 16). According to the above literature summary and our own clinical experience, we consider that a 2-month course may not be enough to treat CM-PIIRS, especially severe cases. Moreover, the course of corticosteroids in CM-PIIRS should be based on clinical, CSF, and brain MRI results.

Due to the decline of immune function, the elderly individuals are often more susceptible to infectious diseases including central nervous system (CNS) infections (22–24). In a study including 99 CM patients, 38.4% (38/99) were elderly CM patients (age >65 years old) which had a high mortality rate (36.8%, 14/38) (25). Therefore, we should pay attention to the treatment of elderly CM patients. In our case, he developed PIIRS and got significant improvement in clinical manifestation and brain MRI after receiving corticosteroids. However, as far as we know, we should use corticosteroids with caution for elderly patients because corticosteroids have some adverse events such as osteopenia and fracture (26, 27). Fortunately, no severe steroid-induced adverse events occurred in this case. In general, the use of corticosteroids in elderly CM-PIIRS patients is probably not only effective but also safe if we closely monitor patients’ conditions.

## Conclusion

PIIRS is an important cause of poor outcome in CM patients. Therefore, it is very important to recognize and actively treat in these patients. Even in elderly patients, corticosteroid treatment is still beneficial. Moreover, 2 months of corticosteroid treatment may be not enough to treat CM-PIIRS.

## Data Availability Statement

The original contributions presented in the study are included in the article/supplementary material. Further inquiries can be directed to the corresponding authors.

## Ethics Statement

The study involving human participants was reviewed and approved by the Medical Ethics Committee of the Third Affiliated Hospital of Sun Yat-sen University. Informed consent was obtained from the individual participant included in the study.

## Author Contributions

YJ and FP were involved in the literature review, planning, and writing of the manuscript. JyL, JL, B-eQ, SY, AW, LY, ZS, and XX collected the data. YJ and FP were involved in the case identification, literature review, and planning and editing of the manuscript. YJ, FP, JyL, and JL were involved in the planning, writing, and editing of the manuscript. All authors contributed to the article and approved the submitted version.

## Funding

This work is supported by grants from the Natural Science Foundation of China (82071265) and Guangzhou Science and Technology Planning Project (202102010288).

## Conflict of Interest

The authors declare that the research was conducted in the absence of any commercial or financial relationships that could be construed as a potential conflict of interest.

## Publisher’s Note

All claims expressed in this article are solely those of the authors and do not necessarily represent those of their affiliated organizations, or those of the publisher, the editors and the reviewers. Any product that may be evaluated in this article, or claim that may be made by its manufacturer, is not guaranteed or endorsed by the publisher.

## Abbreviations

CM, cryptococcal meningitis; PIIRS, post-infectious inflammatory response syndrome; ART, antiretroviral therapy; IRIS, immune reconstitution inflammatory syndrome; GCS, Glasgow Coma Scale; CSF, cerebrospinal fluid; OP, opening pressure; WCC, white cell count; CRAG, cryptococcal antigen titer; LP, lumbar puncture; CST, corticosteroid salvage therapy.

## References

[1] Jarvis JN Meintjes G Williams A Brown Y Crede T Harrison TS. Adult Meningitis in a Setting of High HIV and TB Prevalence: Findings From 4961 Suspected Cases. BMC Infect Dis (2010) 10:67. doi: 10.1186/1471-2334-10-67 20230635 PMC3161361

[2] Park BJ Wannemuehler KA Marston BJ Govender N Pappas PG Chiller TM. Estimation of the Current Global Burden of Cryptococcal Meningitis Among Persons Living With HIV/AIDS. AIDS (2009) 23(4):525–30. doi: 10.1097/QAD.0b013e328322ffac 19182676

[3] Dromer F Mathoulin-Pelissier S Launay O Lortholary O. French Cryptococcosis Study G. Determinants of Disease Presentation and Outcome During Cryptococcosis: The CryptoA/D Study. PLoS Med (2007) 4(2):e21. doi: 10.1371/journal.pmed.0040021 17284154 PMC1808080

[4] Haddow LJ Colebunders R Meintjes G Lawn SD Elliot JH Manabe YC. Cryptococcal Immune Reconstitution Inflammatory Syndrome in HIV-1-Infected Individuals: Proposed Clinical Case Definitions. Lancet Infect Dis (2010) 10(11):791–802. doi: 10.1016/S1473-3099(10)70170-5 21029993 PMC3026057

[5] Williamson PR Jarvis JN Panackal AA Fisher MC Molloy SF Loyse A. Cryptococcal Meningitis: Epidemiology, Immunology, Diagnosis and Therapy. Nat Rev Neurol (2017) 13(1):13–24. doi: 10.1038/nrneurol.2016.167 27886201

[6] Anjum S Dean O Kosa P Magone MT King KA Fitzgibbon E. Outcomes in Previously Healthy Cryptococcal Meningoencephalitis Patients Treated With Pulse - Taper Corticosteroids for Post-Infectious Inflammatory Syndrome. Clin Infect Dis (2020) 73(9):e2789–98. doi: 10.1093/cid/ciaa1901 PMC8563180 33383587

[7] Williamson PR. Post-Infectious Inflammatory Response Syndrome (PIIRS): Dissociation of T-Cell-Macrophage Signaling in Previously Healthy Individuals With Cryptococcal Fungal Meningoencephalitis. Macrophage (Houst) (2015) 2:e1078. doi: 10.14800/Macrophage.1078 27064474 PMC4825797

[8] Anjum S Williamson PR. Clinical Aspects of Immune Damage in Cryptococcosis. Curr Fungal Infect Rep (2019) 13(3):99–108. doi: 10.1007/s12281-019-00345-7 33101578 PMC7580832

[9] Decote-Ricardo D Larocque-De-Freitas IF Rocha JDB Nascimento DO Nunes MP Morrot A. Immunomodulatory Role of Capsular Polysaccharides Constituents of Cryptococcus Neoformans. Front Med (Lausanne) (2019) 6:129. doi: 10.3389/fmed.2019.00129 31275938 PMC6593061

[10] Liu J Luo C Li M Wang Y-J Xu X Yang L. Predictors of Postinfectious Inflammatory Response Syndrome in HIV-Negative Immunocompetent Cryptococcal Meningitis. J Neurol Neurosurg Psychiatry (2020) jnnp-2020-324921. doi: 10.1136/jnnp-2020-324921 33277354

[11] Yang Y Li M Yang L Tian Q Qin B. Clinical, Radiographic Features and Long-Term Outcomes of Paradoxical Cryptococcosis-Associated Immune Reconstitution Inflammatory Syndrome Secondary to the Ventriculoperitoneal Shunt. J Infect (2021) 83(5):607–35. doi: 10.1016/j.jinf.2021.08.025 34419560

[12] Pirofski LA Casadevall A. Immune-Mediated Damage Completes the Parabola: Cryptococcus Neoformans Pathogenesis Can Reflect the Outcome of a Weak or Strong Immune Response. mBio (2017) 8(6):e02063–17. doi: 10.1128/mBio.02063-17 PMC5727418 29233901

[13] Romani L Williamson PR Di Cesare S Matteo GD De Luca M Carsetti R. Cryptococcal Meningitis and Post-Infectious Inflammatory Response Syndrome in a Patient With X-Linked Hyper IgM Syndrome: A Case Report and Review of the Literature. Front Immunol (2021) 12:708837. doi: 10.3389/fimmu.2021.708837 34335625 PMC8320724

[14] Liu J Li M Gan ZQ Wang Y-J Lin C-R Chen Z-L. Postinfectious Inflammatory Response Syndrome in HIV-Uninfected and Nontransplant Men After Cryptococcal Meningitis. Future Microbiol (2020) 15:613–21. doi: 10.2217/fmb-2019-0252 32490698

[15] Cheng JH Cheema R Williamson PR Dimitriades VR. Case Report: Paradoxical Inflammatory Response Syndrome in a Previously Healthy, HIV-Negative, Pediatric Patient With Cryptococcus Gatii Meningitis. Front Pediatr (2021) 9:703895. doi: 10.3389/fped.2021.703895 34513762 PMC8424186

[16] Tanu S Mihir M Rajeev S Annu A. Neurological Worsening During Treatment of an Immunocompetent Adult With Cryptococcus Neoformans Meningitis. Med Mycol Case Rep (2020) 27:48–51. doi: 10.1016/j.mmcr.2020.01.001 31993318 PMC6976910

[17] Kathiresu R Zinat SN Fernando M. Immune Reconstitution Inflammatory Syndrome Following Cryptococcal Neoformans Infection in an Immunocompetent Host: A Case Report and Review of the Literature. IDCases (2020) 19:e00699. doi: 10.1016/j.idcr.2020.e00699 32055440 PMC7005434

[18] Kulkarni A Philip VJ Varghese GK Nagendra CV. Cryptococcal Postinfectious Inflammatory Response Syndrome in an Immunocompetent Host. Ann Indian Acad Neurol (2019) 22(3):322–4. doi: 10.4103/aian.AIAN_29_18 PMC6613424 31359947

[19] Mehta GU Panackal AA Murayi R Bennett JE Williamson PR Chittiboina P. Corticosteroids for Shunted Previously Healthy Patients With Non-HIV Cryptococcal Meningoencephalitis. J Neurol Neurosurg Psychiatry (2018) 89(2):219–20. doi: 10.1136/jnnp-2017-315830 PMC5702587 28550070

[20] Panackal AA Wuest SC Lin YC Wu T Zhang N Kosa P. Paradoxical Immune Responses in Non-HIV Cryptococcal Meningitis. PLoS Pathog (2015) 11(5):e1004884. doi: 10.1371/journal.ppat.1004884 26020932 PMC4447450

[21] Perfect JR Dismukes WE Dromer F Goldman DL Graybill JR Hamill RJ. Clinical Practice Guidelines for the Management of Cryptococcal Disease: 2010 Update by the Infectious Diseases Society of America. Clin Infect Dis (2010) 50(3):291–322. doi: 10.1086/649858 20047480 PMC5826644

[22] Werner H Kuntsche J. Infection in the Elderly–What Is Different? Z Gerontol Geriatr (2000) 33(5):350–6. doi: 10.1007/s003910070031 11130188

[23] Liang SY Mackowiak PA. Infections in the Elderly. Clin Geriatr Med (2007) 23(2):441–456, viii. doi: 10.1016/j.cger.2007.01.010 17462528

[24] Lai WA Chen SF Tsai NW Chang C-C Chang W-N Lu C-H. Clinical Characteristics and Prognosis of Acute Bacterial Meningitis in Elderly Patients Over 65: A Hospital-Based Study. BMC Geriatr (2011) 11:91. doi: 10.1186/1471-2318-11-91 22204457 PMC3282677

[25] Tsai WC Lien CY Lee JJ Hsiao W-C Huang C-R Tsai N-W. The Clinical Characteristics and Therapeutic Outcomes of Cryptococcal Meningitis in Elderly Patients: A Hospital-Based Study. BMC Geriatr (2019) 19(1):91. doi: 10.1186/s12877-019-1108-0 30909914 PMC6434878

[26] Hahn TJ. Corticosteroid-Induced Osteopenia. Arch Intern Med (1978) 138 Spec No:882–5. doi: 10.1001/archinte.1978.03630300050010 646579

[27] Adinoff AD Hollister JR. Steroid-Induced Fractures and Bone Loss in Patients With Asthma. N Engl J Med (1983) 309(5):265–8. doi: 10.1056/NEJM198308043090502 6866051

